# Squamous Odontogenic Tumor: A Systematic Review

**DOI:** 10.1111/odi.70340

**Published:** 2026-04-24

**Authors:** Paula Sousa Lopes de Cascaes, Licia Pacheco Teixeira, Andresa Borges Soares, Izabel Regina Fischer Rubira‐Bullen, Monikelly Carmo Narchini do Nascimento, José Luiz Cintra Junqueira, Mariana Quirino Silveira Soares

**Affiliations:** ^1^ Division of Oral Radiology Faculdade São Leopoldo Mandic Campinas Brazil; ^2^ Department of Surgery, Stomatology, Pathology and Radiology, Bauru School of Dentistry University of São Paulo Bauru Brazil; ^3^ Division of Oral Radiology São Leopoldo Mandic Campinas Brazil

**Keywords:** histopathology, Jaw neoplasms, odontogenic tumors, squamous odontogenic tumor, systematic review

## Abstract

**Objective:**

Squamous odontogenic tumor is a rare benign epithelial odontogenic neoplasm whose overlapping features with other odontogenic lesions may hinder diagnosis and management. This systematic review analyzed clinical, radiographic, histopathological characteristics and treatment outcomes of SOT.

**Methods:**

Comprehensive search was conducted in April 2025 across PubMed, Web of Science, Scopus, Embase, Cochrane Library, Livivo, BVS, SciELO, ProQuest, OpenGrey, IBICT/BDTD, and Google Scholar. Two independent reviewers performed study selection, data extraction, and risk‐of‐bias assessment. Only cases fulfilling diagnostic criteria established by the WHO Classification of Head and Neck Tumors (2024) were included. Cases descriptions and images were assessed by an experienced oral pathologist to confirm diagnostic consistency.

**Results:**

A total of 65 SOT lesions were identified, including 59 intraosseous and 6 peripheral cases. Intraosseous lesions occurred across a wide age range with slight male predominance. Most lesions were solitary and located in the maxilla or posterior jaw regions. Radiographically, intraosseous lesions were predominantly unilocular, with triangular configuration most frequently reported. Most lesions were treated with conservative surgery, particularly surgical excision, and recurrence occurred in a minority of intraosseous cases. Peripheral lesions were solitary and showed a benign course without recurrence.

**Conclusion:**

SOT generally presents as a benign lesion with a favorable prognosis.

## 1 Introduction

Odontogenic tumors are a diverse group of neoplasms originating from tissues involved in odontogenesis. Their biological behavior can range from indolent to aggressive. The clinical, radiographic, and histopathological characteristics of the rare benign epithelial entity known as squamous odontogenic tumor (SOT) remain incompletely understood. In addition, its similarity to other odontogenic lesions may impair an accurate diagnosis (Buchner et al. 2006; Rath et al. 2016; WHO 2024).

Originally described in 1975 by Pullon, SOT has been classified by the WHO (2024) since 2005 as a benign epithelial odontogenic tumor due to its slow growth and squamous differentiation (Pullon et al. 1975). Its essential diagnostic criteria are location in dentate areas of the mandible or maxilla, well‐differentiated squamous epithelium in a fibrous or myxoid stroma, without significant keratinization and absence of peripheral palisading, stellate reticulum, cellular atypia or pleomorphism (WHO 2024).

Although often asymptomatic, some SOT lesions can lead to pain, tooth mobility, and/or bone expansion. Radiographically, these lesions typically appear as triangular‐ or semicircular‐shaped unilocular radiolucencies with well‐defined margins and may eventually cause root displacement (Badni et al. 2012). The usual treatment for SOT is conservative surgical excision, and the recurrence rate for unifocal lesions is low (Pullon et al. 1975).

SOT presumably originates from epithelial remnants of Malassez of the periodontal ligament or the basal layer of the oral mucosa. Evidence indicates that specific molecular pathways such as NOTCH receptors and their ligands, as well as mutations in the ameloblastin gene (AMBN), are involved in SOT pathogenesis (Siar et al. 2010; Perdigão et al. 2004). Reports of SOT lesions involving family members support the hypothesis of hereditary predisposition (Leider et al. 1989; Croonenborghs et al. 2020).

However, the scattered information from SOT case reports limits the establishment of evidence‐based diagnostic and therapeutic approaches (Pullon et al. 1975; Goldblatt et al. 1982; Aborisade and Soyele 2019). There are limited studies that have critically evaluated the clinical, imaging, histological, and therapeutic findings of these lesions (Chrcanovic and Gomez 2018; Upadhyaya et al. 2020). Therefore, this systematic review aimed to comprehensively analyze the available evidence on clinical, radiographic, and histopathological characteristics, as well as therapeutic outcomes of SOT lesions.

## 2 Materials and Methods

This systematic review was registered at the International Prospective Register of Systematic Reviews (PROSPERO—file number CRD 42024605302) and followed the Preferred Reporting Items for Systematic Reviews and Meta‐Analyses Protocols (PRISMA) (Page et al. 2021).

### 2.1 Eligibility Criteria

Eligibility criteria were established according to the WHO 2024 classification. Data extraction was performed in a structured manner, and the individual risk of bias was assessed using the Joanna Briggs Institute checklist. Clinical, radiographic, histopathological, and therapeutic data were analyzed on a case‐by‐case basis and organized in Supporting Material as a standardized and accessible dataset (Data S1).

The PECO (Population, Exposure, Control, Outcome) framework was applied to formulate the research question: (P) patients with SOT; (E) diagnosis confirmed by biopsy; (C) not applicable; and (O) clinical, radiographic, and histopathological characteristics, treatment modalities, and recurrence.

Eligible studies included descriptive designs (prospective or retrospective), case series, and case reports describing SOT cases that fulfilled the diagnostic criteria established by the WHO. According to the WHO Classification of Head and Neck Tumors (2024), SOT occurs in dentate areas of the jaws and is characterized histologically by variably sized and shaped islands of well‐differentiated squamous epithelium within a fibrous or myxoid stroma. These epithelial islands show uniform squamous differentiation without significant keratinization and lack peripheral palisading or stellate reticulum. The peripheral cells are typically flat to cuboidal with rare mitotic figures, whereas the central cells may exhibit cystic degeneration, individual cell keratinization, and occasional calcification. Minor histopathological changes such as mucous metaplasia, sebaceous differentiation, or ghost cell–like areas may also be observed. Importantly, SOT does not demonstrate peripheral palisading, reverse nuclear polarity, cellular atypia, or pleomorphism.

In addition, only cases presenting histopathological microphotographs of sufficient quality to allow proper morphological assessment were included. The images were required to demonstrate adequate resolution and magnification to permit independent evaluation by an oral and maxillofacial pathologist.

All cases were individually assessed by a pathologist with 20 years of experience (ABS) who considered both the case description and the figures to ensure diagnostic consistency according to the current WHO (2024) criteria. In reports describing both incisional and excisional biopsies, only the final histopathological diagnosis obtained from the definitive (excisional) specimen was considered for analysis. Cases reporting synchronous tumors in the same anatomical locus were excluded, as this condition could introduce diagnostic ambiguity and potentially compromise data reliability.

SOT‐like epithelial proliferations occurring in the lining of odontogenic cysts, which are recognized by the WHO as close histological mimics of SOT, were excluded. No restrictions were applied regarding publication date, language, or follow‐up period. Books, book chapters, editorials, conference abstracts, and narrative reviews were excluded.

### 2.2 Information Sources

In April 2025, studies were searched in PubMed, Scopus, Web of Science, SciELO, Cochrane Library, Embase, and LIVIVO databases. Other sources included ProQuest Open Gray, IBICT/BDT, and Google Scholar (first 101 results). Records were also manually searched on the reference lists of the included studies.

### 2.3 Search Strategy and Study Selection

Health sciences descriptors (DeCS) and medical subject headings (MeSH) “Squamous odontogenic tumor” were combined with the Boolean operators “AND” and “OR” to create the search strategies (Data S1). Duplicates were excluded using reference management software (EndNote Web, Clarivate Analytics, USA). The records were then exported to web‐based software for systematic reviews (Rayyan, Qatar Computing Research Institute, Qatar) (Ouzzani et al. 2016). Two reviewers (PSLC and LPT) independently selected the studies by reading titles and abstracts. The same eligibility criteria were applied during full‐text assessment. Disagreements were resolved by a third reviewer (MQSS).

### 2.4 Data Collection

Two reviewers (PSLC and MQSS) independently gathered the following data: authors, publication year, country, gender, age, ethnicity, symptoms, evolution time, clinical characteristics, pain, location, size, radiographic characteristics, histopathological characteristics, diagnostic methods, treatment, follow‐up, and recurrence. Disagreements were resolved by a third reviewer (MCNN). The reviewers were previously calibrated during a pilot study involving data from 10 studies.

### 2.5 Individual Risk of Bias

Two reviewers (PSLC and MQSS) independently assessed the bias risk and methodological quality of each study, and any disagreements were resolved by a third reviewer (ABS). The Joanna Briggs Institute Critical Appraisal Tool for case reports (JBI Checklist for Case Reports) was used to address the following questions: (Q1) Were the patient's demographic characteristics clearly described? (Q2) Was the patient's history clearly described and presented as a timeline? (Q3) Was the current clinical condition on presentation clearly described? (Q4) Were diagnostic tests or assessment methods and results clearly described? (Q5) Was the intervention(s) or treatment procedure(s) clearly described? (Q6) Was the post‐intervention clinical condition clearly described? (Q7) Were adverse events (harms) or unanticipated events identified and described? (Q8) Does the case report provide takeaway lessons? Not available or unclear information was considered as a “no” answer. The risk of bias for each study was assessed by the percentage of “yes” answers: high (up to 49%); moderate (between 50% and 69%); or low (over 70%). Studies with more than one case that lacked information for any case were scored “no”.

### 2.6 Measures of Effect

Mean age (years), largest diameter of the lesion (mm), evolution time (months), and recurrence (months) were used as measures of effect. Data extraction was performed in a structured manner. Clinical, radiographic, histopathological, and therapeutic variables were analyzed on a case‐by‐case basis, and studies reporting multiple cases contributed more than one observation to the dataset. Radiographic and histopathological characteristics were summarized using frequencies and proportions. Missing information was recorded as “not reported” and excluded from the denominator when calculating proportions. Effect measures included means and standard deviations for continuous variables with symmetric distributions, while variables with asymmetric distributions were described using medians. Categorical variables were presented as absolute and relative frequencies (Stata version 18.0; StataCorp LLC, College Station, TX, USA).

### 2.7 Summary of Results

Data extracted from the included studies were summarized descriptively in tables, considering the heterogeneity of clinical, radiographic, and histopathological characteristics, treatment, and recurrence. When time intervals were reported as ranges, the midpoint value was used for statistical analysis. Time intervals originally reported in weeks were converted to months for analysis (1 month ≈ 4 weeks). Recurrence rates and time intervals for lesion evolution were calculated based on the available data and reported in absolute and percentage terms. A meta‐analysis was not performed due to the heterogeneity of the data and the observational nature of the included studies.

## 3 Results

### 3.1 Study Selection

After an initial screening of 797 records retrieved from PubMed, Web of Science, Cochrane Library, SciELO, Embase, LIVIVO, Scopus and the BVS, 280 duplicates and 436 records that did not meet the eligibility criteria were excluded. Four records were not available online or at academic libraries, and the respective authors did not respond to requests for access (Fay et al. 1981; Kangvonkit et al. 1981; Yoris et al. 2009; Pucci 2009). In addition, one record (Adebiyi et al. 2006) was not available for full‐text assessment.

After full‐text assessment, 11 studies did not meet the eligibility criteria because they did not fulfill the diagnostic criteria for SOT established by the WHO (2024) (Van Der Waal et al. 1980; Anneroth and Hansen 1982; Swan and McDaniel 1983; Mills et al. 1986; Schwartz‐Arad et al. 1990; Saxby et al. 1993; Beovide and Kornecki 1994; Ruhin et al. 2007; Krithika et al. 2007; Ide et al. 2009; Tarsitano et al. 2012).

Two additional studies were excluded because they reported synchronous tumors occurring in the same anatomical locus, which could introduce diagnostic ambiguity and compromise data reliability (Koshy et al. 2020; Thomas et al. 2021). Furthermore, the study by Shimoyama et al. (1995) was excluded because the lesion described represents a mural SOT‐like epithelial proliferation arising within the wall of an odontogenic cyst, rather than a true SOT. The studies by Alonso‐Juarranz et al. (2024) and Pardhe et al. (2021) were also excluded because, although the lesions were initially reported as SOT, independent evaluation of the histopathological microphotographs suggested features consistent with a malignant neoplasm.

Seven studies were excluded due to insufficient quality of histopathological microphotographs, as the images did not present adequate resolution or magnification to allow reliable evaluation (de Oliveira et al. 2007; Emmanuel et al. 2012; Singh et al. 2014; Tu et al. 2018; Aborisade and Soyele 2019; Oley et al. 2022; Saxena et al. 2022).

In studies reporting multiple cases, each case was individually reassessed according to the WHO (2024) diagnostic criteria. Following this reassessment, case 3 from Doyle et al. (1977) and case 2 from Leventon et al. (1981) were excluded because they represented SOT‐like epithelial proliferations arising in the lining of odontogenic cysts rather than true SOT.

Additionally, case 3 reported by Leider et al. (1989) was excluded from the final dataset because this patient had been previously described in the literature by McNeill et al. (1980). To avoid duplicate case reporting and potential double counting of the same patient, the case was considered only in its original publication (McNeill et al. 1980). The remaining cases from Doyle et al. (1977), Leventon et al. (1981), and Leider et al. (1989) fulfilled the WHO (2024) diagnostic criteria for SOT and were therefore included in the final analysis.

In the case reported by Norris et al. (1984), the patient presented with three lesions, two located in the maxilla and one in the mandible. The mandibular lesion was diagnosed as squamous cell carcinoma (SCC). However, there was no prior histopathological confirmation that this lesion had originated from a SOT. Therefore, only the two maxillary lesions with histopathological features consistent with SOT were included in the present review.

Eight studies were excluded because, after independent evaluation of the histopathological microphotographs, the morphological features were not consistent with the diagnosis of SOT as reported by the authors (Tamaki et al. 1990; Krithika et al. 2007; Kim et al. 2007; Agostini et al. 2011; Beena et al. 2019; Sihmar et al. 2021; Prabhu et al. 2023; Sugianto et al. 2024). When duplicate publications were identified, the most complete and original report was retained for data extraction.

After applying all eligibility criteria, 45 studies were included in the final review (Pullon et al. 1975; Doyle et al. 1977; McNeill et al. 1980; Hopper et al. 1980; Carr et al. 1981; Leventon et al. 1981; Goldblatt et al. 1982; Cataldo et al. 1983; Norris et al. 1984; Kristensen et al. 1985; Monteil and Terestri 1985; Warnock et al. 1985; Hietanen et al. 1985; Tatemoto et al. 1989; Leider et al. 1989; Reichart and Philipsen 1990; Loyola et al. 1990; Yaacob 1990; Baden et al. 1993; Zak and Glickman 1995; Favia et al. 1997; Stoll et al. 1997; Kusama et al. 1998; Ide et al. 1999; Gómez et al. 2001; Haghighat et al. 2002; Barrios et al. 2004; Cillo Jr et al. 2005; Siar et al. 2010; Jones et al. 2011; Badni et al. 2012; Malathi et al. 2012; Bansal and Joshi 2013; Mohr et al. 2015; Lúcio et al. 2015; Anjana et al. 2016; Elmuradi et al. 2017; Verhelst et al. 2018; Mohanty et al. 2019; Laungani et al. 2020; Upadhyaya et al. 2020; Croonenborghs et al. 2020; Cunha et al. 2023; Prasanth et al. 2023; Alotaibi et al. 2024).

Only 60 out of 435 records screened from other sources (OpenGrey, ProQuest, IBICT/BDTD, and Google Scholar and reference lists) met the eligibility criteria. Three records were not available online or at academic libraries, and the respective authors did not respond to requests for access (Sampaio and Teles 1981; Uchida et al. 2008; Reddy et al. 2011). Since 57 of these records had already been identified through the database searches, the remaining studies were assessed through full‐text evaluation.

After full‐text reading, two studies did not meet the eligibility criteria because they did not fulfill the diagnostic criteria for SOT established by the WHO (2024) (Saxby et al. 1993; Takagi et al. 1999). Two additional studies were excluded due to the absence of histopathological microphotographs, which prevented adequate morphological assessment (Pant and Pathak 2013; Archana et al. 2017). Furthermore, one study was excluded because the available histopathological images were not sufficient to support a definitive diagnosis of SOT after independent evaluation (Rath et al. 2016), and another study was excluded due to insufficient image quality for reliable morphological analysis (De Angelis et al. 2023).

After applying all eligibility criteria, an additional five studies obtained from other sources were included in this review (Ikai et al. 2001; Iwaki et al. 2007; Tamgadge et al. 2007; Moussa et al. 2013; Endo et al. 2024), bringing the total to 50 studies included in this review (Data S2).

### 3.2 Characteristics of the Included Studies

All 50 included studies were case reports or series published between 1975 and 2024, which reported 59 intraosseous SOT (90.7%) and 6 peripheral SOT (9.3%). Twenty‐two studies (44%) were originally from the United States of America (Pullon et al. 1975; Doyle et al. 1977; McNeill et al. 1980; Hopper et al. 1980; Carr et al. 1981; Leventon et al. 1981; Goldblatt et al. 1982; Cataldo et al. 1983; Norris et al. 1984; Warnock et al. 1985; Leider et al. 1989; Zak and Glickman 1995; Gómez et al. 2001; Haghighat et al. 2002; Barrios et al. 2004; Cillo Jr et al. 2005; Jones et al. 2011; Elmuradi et al. 2017; Laungani et al. 2020; Upadhyaya et al. 2020; Alotaibi et al. 2024; Endo et al. 2024), 07 studies (14%) from India (Tamgadge et al. 2007; Badni et al. 2012; Malathi et al. 2012; Bansal and Joshi 2013; Anjana et al. 2016; Mohanty et al. 2019; Prasanth et al. 2023), 6 studies (12%) from Japan (Tatemoto et al. 1989; Kusama et al. 1998; Ide et al. 1999; Ikai et al. 2001; Iwaki et al. 2007; Siar et al. 2010), 3 studies (6%) from Brazil (Loyola et al. 1990; Lúcio et al. 2015; Cunha et al. 2023), 3 studies (6%) from Germany (Reichart and Philipsen 1990; Stoll et al. 1997; Mohr et al. 2015), 2 studies (4%) from France (Monteil and Terestri 1985; Baden et al. 1993), 2 studies (4%) from Belgium (Verhelst et al. 2018; Croonenborghs et al. 2020), and one study (2%) from each of the following countries: Denmark (Kristensen et al. 1985), Finland (Hietanen et al. 1985), Italy (Favia et al. 1997), Malaysia (Yaacob 1990) e Egypt (Moussa et al. 2013).

### 3.3 Risk of Bias

A total of 39 (78%), 10 (20%), and 1 (2%) studies respectively presented low, moderate, and high risk of bias (Data S3). Q1 was considered positive only when age, gender, and ethnicity data were reported. Patient ethnicity was reported in 27 studies (54%) (Pullon et al. 1975; Doyle et al. 1977; McNeill et al. 1980; Hopper et al. 1980; Carr et al. 1981; Cataldo et al. 1983; Kristensen et al. 1985; Monteil and Terestri 1985; Warnock et al. 1985; Hietanen et al. 1985; Tatemoto et al. 1989; Leider et al. 1989; Loyola et al. 1990; Yaacob 1990; Zak and Glickman 1995; Stoll et al. 1997; Kusama et al. 1998; Gómez et al. 2001; Haghighat et al. 2002; Barrios et al. 2004; Cillo Jr et al. 2005; Tamgadge et al. 2007; Siar et al. 2010; Lúcio et al. 2015; Elmuradi et al. 2017; Croonenborghs et al. 2020; Cunha et al. 2023). Only 1 study (2%) did not report the patient's gender (Favia et al. 1997). The clinical characteristics of the lesions were not detailed in 3 studies (6%) (Goldblatt et al. 1982; Tatemoto et al. 1989; Leider et al. 1989) and symptoms were not reported in 7 studies (14%) (Pullon et al. 1975; Goldblatt et al. 1982; Hietanen et al. 1985; Tatemoto et al. 1989; Baden et al. 1993; Malathi et al. 2012; Elmuradi et al. 2017).

The radiographic characteristics of the lesions were not described in three studies (6%) (Baden et al. 1993; Leventon et al. 1981; Goldblatt et al. 1982). The missing information regarding follow‐up in 15 studies (30%) prevented positive responses to Q6 and Q7 (Doyle et al. 1977; Goldblatt et al. 1982; Norris et al. 1984; Warnock et al. 1985; Tatemoto et al. 1989; Leider et al. 1989; Gómez et al. 2001; Tamgadge et al. 2007; Jones et al. 2011; Badni et al. 2012; Malathi et al. 2012; Moussa et al. 2013; Mohanty et al. 2019; Alonso‐Juarranz et al. 2024; Endo et al. 2024) (Data S3).

### 3.4 Synthesis of Outcomes

The 65 cases were grouped according to lesion location and classified as intraosseous SOT (59 cases) or peripheral SOT (6 cases). The study by Baden et al. (1993) reported three cases of SOT: cases 1 and 2 corresponded to intraosseous lesions, whereas case 3 represented a peripheral lesion. Analyses of clinical, radiographic, and histopathological characteristics, treatments, and prognoses were conducted descriptively and stratified between groups (Tables 1 and 2 and Data S4 and S5).

**TABLE 1:** Summary of characteristics of intraosseous SOT lesions.

Characteristics^α^			*n*	%
Patient's gender (*n* = 58)	Male		30	51.7
	Female		28	48.3
Lesion number (*n* = 59)	Single		50	84.7
	Multiple		9	15.3
Anatomical site (*n* = 58)	Maxilla		29	50
	Mandible		20	34.5
	Multifocal		9	15.5
Anteroposterior distribution (*n* = 59)	Anterior		18	30.5
	Posterior		29	49.2
	Anterior and posterior		3	5.1
Side (*n* = 27)	Right		15	55.6
	Left		12	44.4
Pain (*n* = 46)	Absence		38	82.6
	Presence		8	17.4
Clinical characteristics (*n* = 57)	Swelling		22	38.6
	Swelling and tooth mobility		8	14
	Tooth mobility		8	14
	Asymptomatic		6	10.5
	Swelling and sensitivity		2	3.5
	Swelling and ulceration		2	3.5
	Gingival recession		2	3.5
	Pain (isolated)		2	3.5
	Swelling + paresthesia		1	1.8
	Pain + drainage + tenderness to percussion		1	1.8
	Thermal sensitivity		1	1.8
	Discomfort		1	1.8
	Fistula + trismus		1	1.8
Radiographic characteristics	Shape (*n* = 19)	Irregular	2	10.5
		Triangular	10	52.6
		Ovoid/oval	2	10.5
		Hemispherical	2	10.5
		Pear‐shaped/scalloped	1	5.3
		Band‐like	1	5.3
		Teardrop	1	5.3
	Locularity (*n* = 14)	Unilocular	10	71.4
		Multilocular	4	28.6
		Impact tooth	9	19.1
	Findings	Root resorption	3	6.4
		Root divergence	13	27.7
Histopathological characteristics	Microcystic degeneration		45	76.3
	Calcification		20	33.9
	Keratinization		16	27.1
Treatment (*n* = 58)	Surgical excision		22	37.9
	Surgical excision + curettage		8	13.8
	Surgical excision + tooth extraction		9	15.5
	Surgical excision + tooth extraction + curettage		1	1.7
	Tooth extraction + curettage		5	8.6
	Curettage		1	1.7
	Bone resection		2	3.4
	Bone resection + tooth extraction		6	10.3
	Bone resection + surgical excision		3	5.2
	Bone resection + tooth extraction + curettage		1	1.7
Recurrence (*n* = 41)	Absence		35	85.4
	Presence		6	14.6
Follow‐up (*n* = 41)	Absence		2	4.9
	Presence		39	95.1
	**Mean** ^a^		**Median (range)**	
Age (years)	36.8		37 (10–67)	
Evolution time (months)	11		4.25 (0.75–60)	
Diameter (mm)	24.6		20 (2–70)	
Follow‐up (months)	32.3		15 (1–216)	

*Note:* α Only data reported as “absence” or “presence” were included (missing information was excluded).

^a^ Values are presented as mean and median (range) and were calculated from the available data.

**TABLE 2:** Summary of characteristics of peripheral SOT lesions.

Characteristics^α^		*n*	%
Patient's gender (*n* = 6)	Male	4	66.7
	Female	2	33.3
Lesion number (*n* = 6)	Single	6	100
	Multiple	0	0
Anatomical site (*n* = 6)	Maxilla	3	50
	Mandible	3	50
	Multifocal	0	0
Anteroposterior distribution (*n* = 5)	Anterior	3	60
	Posterior	2	40
	Anterior and posterior	0	0
Side (*n* = 2)	Right	1	50
	Left	1	50
Pain (*n* = 3)	Absence	2	66.7
	Presence	1	33.3
Clinical characteristics (*n* = 57)	Swelling	5	83.3
	Pain	1	16.7
Histopathological characteristics	Microcystic degeneration	5	83.3
	Calcification	1	33.3
	Keratinization	2	33.3
Treatment (*n* = 6)	Surgical excision	5	83.3
	Tooth extraction and surgical excision	1	16.7
Recurrence (*n* = 5)	Absence	5	100
	Presence	0	0
Follow‐up (*n* = 5)	Absence	5	100
	Presence	0	0
	**Mean** ^a^	**Median (range)**	
Age (years)	45.5	49 (11–74).	
Evolution time (months)	20	24 (12–24)	
Diameter (mm)	—	Not applicable	
Follow‐up (months)	10	12 (5–15)	

*Note:* α Only data reported as “absence” or “presence” were included (missing information was excluded).

^a^ Values are presented as mean and median (range) and were calculated from the available data.

### 3.5 Intraosseous SOT

Patients with intraosseous SOT ranged in age from 10 to 67 years, with a mean age of 36.8 years and a median age of 37 years. Patient sex was reported for 58 cases, showing a slight male predominance (51.7%), while 48.3% were female. Only one case did not report patient sex (Favia et al. 1997). Ethnicity was reported in 57.6% of cases. Among these, Caucasian patients represented 28.8%, followed by Afroamerican patients (22.0%), Asian patients (5.1%), and one Turkish patient (1.7%), while 42.4% of cases did not report ethnicity. Ethnicity categories were recorded as reported in the original studies.

Solitary lesions accounted for 84.7% of the reports, whereas multiple lesions were observed in 15.3%. Regarding anatomical site, the maxilla was the most frequently affected location (50.0%), followed by the mandible (34.5%). Concerning the anteroposterior distribution, lesions occurred predominantly in the posterior region (49.2%), whereas three cases involved both anterior and posterior regions, corresponding to lesions extending from the anterior region toward one side of the jaws. Among these, one extended to the right (Doyle et al. 1977), one to the left (Pullon et al. 1975), and one did not specify the side of extension (Goldblatt et al. 1982). Analysis of laterality showed that lesions were slightly more frequent on the right side (55.6%) than on the left (44.4%). Multifocal lesions were excluded from the analyses of anatomical site, anteroposterior distribution, and laterality to avoid duplication of anatomical locations. Multifocal involvement was reported in nine cases (Pullon et al. 1975; McNeill et al. 1980; Norris et al. 1984; Hopper et al. 1980; Leider et al. 1989; Elmuradi et al. 2017; Verhelst et al. 2018; Croonenborghs et al. 2020).

Clinical characteristics were described in 57 cases. Two cases reported by Goldblatt et al. (1982) did not present information regarding clinical symptoms and were therefore not considered in the analysis of specific clinical manifestations. Swelling was the most frequently reported clinical finding (38.6%). Isolated tooth mobility was observed in 14.0% of cases. Swelling associated with tooth mobility was also reported in 14.0% of cases. Asymptomatic lesions accounted for 10.5% of cases and were generally identified as incidental findings during radiographic examination (Pullon et al. 1975; Leventon et al. 1981; Warnock et al. 1985; Leider et al. 1989; Gómez et al. 2001; Cillo Jr et al. 2005). Sensitivity associated with swelling was observed in 3.5% of cases (Mohr et al. 2015; Laungani et al. 2020), while swelling with ulceration was also reported in 3.5% (Baden et al. 1993; Iwaki et al. 2007). Gingival recession was described in 3.5% of cases (Favia et al. 1997; Haghighat et al. 2002). Less frequent clinical manifestations included swelling associated with paresthesia (1.8%) (Monteil and Terestri 1985), pain associated with drainage and tenderness to percussion (1.8%) (Goldblatt et al. 1982), isolated thermal sensitivity (1.8%) (Cataldo et al. 1983), discomfort (1.8%) (Goldblatt et al. 1982), and fistula associated with trismus (1.8%) (Alotaibi et al. 2024). In two cases (3.5%), pain was reported as the only clinical manifestation of the tumor (McNeill et al. 1980; Upadhyaya et al. 2020). After excluding these cases from the comparative analysis, pain associated with other clinical manifestations was reported in 17.4% of the cases. Information regarding the presence or absence of pain was not available in 11 cases. The median evolution time and lesion diameter were 4.25 months and 20 mm, respectively.

Radiographically, all lesions described in the available reports were characterized as radiolucent. Regarding lesion morphology, the triangular configuration was the most frequent (52.6%), followed by irregular (Doyle et al. 1977; Hopper et al. 1980), ovoid/oval (Goldblatt et al. 1982; Monteil and Terestri 1985), and hemispherical patterns (Goldblatt et al. 1982; Yaacob 1990) (10.5% each). Less frequently reported morphologies included pear‐shaped/scalloped (Cataldo et al. 1983), band‐like (Upadhyaya et al. 2020), and teardrop‐shaped (Laungani et al. 2020) patterns (5.3% each). Concerning locularity, most lesions presented a unilocular pattern (71.4%), whereas 28.6% were described as multilocular. With respect to effects on adjacent structures, impacted teeth were observed in 19.1% of cases, root resorption in 6.4%, and root divergence in 27.7%. As an additional radiographic finding, displacement of the mandibular canal was reported by Stoll et al. (1997). These radiographic findings were recorded only when explicitly described in the original reports. Multifocal cases were excluded from the radiographic morphology analysis because the presence of multiple lesions with distinct radiographic presentations in the same patient could lead to duplication or misclassification of radiographic characteristics. Additionally, information regarding these alterations was not reported in two cases (Siar et al. 2010; Goldblatt et al. 1982), while one case explicitly reported the absence of radiographic alterations (Barrios et al. 2004).

Histopathologically, all cases exhibited islands and strands of well‐differentiated squamous epithelium embedded within a fibrous connective tissue stroma. Additional histopathological features, including microcystic degeneration, calcification, and keratinization, were recorded only when explicitly described in the original reports and were not assumed when not reported. Among these findings, microcystic degeneration was the most frequently reported feature (76.3%), followed by intraepithelial keratinization (33.9%) and calcifications (27.1%). These features represent the most commonly described histopathological variations accompanying the epithelial proliferation characteristic of SOT.

Most cases were managed with conservative surgical approaches. Surgical excision was the most frequently reported treatment, accounting for 37.9% of cases when performed as a single procedure and 69.0% overall when considering combinations with other conservative techniques, including curettage (13.8%), tooth extraction (15.5%), or both tooth extraction and curettage (1.7%). Less frequently, treatment consisted of tooth extraction associated with curettage (8.6%) or isolated curettage (1.7%). Bone resection procedures were reported in 22.4% of cases overall, either as an isolated treatment (3.4%) or combined with tooth extraction (10.3%), surgical excision (5.2%), or tooth extraction and curettage (1.7%). The case reported by Hopper et al. (1980) was not included in the quantitative treatment analysis because different surgical approaches were used for distinct anatomical sites in the same patient, with bone resection associated with tooth extraction for the maxillary lesions and surgical excision for the mandibular lesion. To avoid duplication of treatment data within a single case, this report was excluded from the analysis.

Follow‐up data were available for 67.8% of the reported cases. Among these, 39 cases had a documented follow‐up period, whereas 2 cases did not undergo follow‐up, corresponding to the reports by Norris et al. (1985) and Elmuradi et al. (2017). In the case reported by Norris et al., follow‐up for the SOT could not be assessed because the patient died due to a synchronous mandibular squamous cell carcinoma. In five studies, follow‐up was mentioned but the duration was not specified, including two cases reported by Leider et al. (1989), as well as the reports by Iwaki et al. (2007), Verhelst et al. (2018), and Laungani et al. (2020).

Regarding recurrence, data were available in 69.5% of cases, whereas 18 studies did not report recurrence information. Recurrence was observed in 14.6% of cases (6 cases), reported in Pullon et al. (1975), McNeill et al. (1980), Goldblatt et al. (1982), Ide et al. (1999), Mohr et al. (2015), and Verhelst et al. (2018). The remaining 35 cases (85.4%) showed no recurrence. Twenty‐four studies reported the lesion evolution time and lesion size. The median follow‐up period was 15 months.

Familial involvement of the jaws was reported in two studies (Leider et al. 1989; Croonenborghs et al. 2020). Leider et al. (1989) described three siblings with multicentric SOT; however, one of these cases had been previously reported by McNeill et al. (1980) and was therefore excluded from the present analysis to avoid duplicate case reporting. Croonenborghs et al. (2020) later documented a familial occurrence involving a mother and her son, both presenting with multifocal SOT.

Malignant transformation of SOT into SCC was reported in two studies (Ide et al. 1999; Alotaibi et al. 2024). In both cases, histopathological examination demonstrated areas consistent with SOT associated with epithelial components showing cytological atypia and invasive SCC.

### 3.6 Peripheral SOT

Patients with peripheral SOT ranged in age from 11 to 74 years, with a mean age of 45.5 years and a median age of 49 years. Patient sex was reported for all cases, showing a male predominance (66.7%), while 33.3% were female. Ethnicity was not reported in any of the included cases.

All lesions were solitary. The maxilla and mandible were equally affected (50% each). Concerning the anteroposterior distribution, lesions occurred more frequently in the anterior region (60%), followed by the posterior region (40%). Clinical characteristics were described in all cases. Swelling was the most frequently reported clinical finding (83.3%). Pain was the only clinical manifestation in one case (Prasanth et al. 2023), accounting for 16.7% of cases. Information regarding the presence of pain was not reported in three cases (Baden et al. 1993; Hietanen et al. 1985; Malathi et al. 2012). The mean lesion evolution time was 20 months, with a median of 24 months. Lesion diameter could not be determined, as no measurable radiographic alterations were described in the reports.

Radiographic findings were rarely described in the available reports. In most cases, no radiographic alterations were observed. Loss of interdental bone at the lesion site was reported in one case (Malathi et al. 2012), while an impacted tooth adjacent to the lesion was observed in another case (Prasanth et al. 2023).

Histopathologically, all cases exhibited islands and strands of well‐differentiated squamous epithelium embedded within a fibrous connective tissue stroma. Additional histopathological features were recorded only when explicitly described in the original reports and were not assumed when not reported. Microcystic degeneration was the most frequently reported feature (83.3%), followed by calcifications (33.3%) and keratinization (33.3%). Calcifications were reported in the cases described by Hietanen et al. (1985) and Ikai et al. (2001), whereas keratinization was observed in the cases reported by Malathi et al. (2012) and Hietanen et al. (1985), the latter exhibiting both features.

Most cases were managed with conservative surgical approaches. Surgical excision was the most frequently reported treatment, accounting for 83.3% of cases. In 16.7% of cases, treatment consisted of tooth extraction associated with surgical excision, as reported by Ikai et al. (2001). No other treatment modalities were described in the available reports. Follow‐up data were available for five cases, and no recurrences were reported. The mean follow‐up period was 10 months, with a median of 12 months. Follow‐up information was not reported in one case (Malathi et al. 2012).

No cases of familial involvement of the jaws were reported in the studies describing peripheral SOT.

## 4 Discussion

The present systematic review synthesized the available evidence on the clinical, radiographic, histopathological, and therapeutic characteristics of SOT. Overall, the findings indicate that SOT most frequently presents as an intraosseous lesion affecting a wide age range, with a slight male predominance and a predilection for the maxilla and posterior jaw regions. Radiographically, lesions are most often described as well‐defined radiolucencies, frequently with a triangular configuration between adjacent tooth roots. Conservative surgical management was the most commonly reported treatment, and the overall prognosis was favorable, with recurrence occurring in a minority of intraosseous cases and no recurrences reported among peripheral lesions.

SOT is histopathologically characterized by variably sized and shaped islands and strands of well‐differentiated squamous epithelium embedded within a fibrous or occasionally myxoid connective tissue stroma. The epithelial islands show uniform squamous differentiation, typically composed of flat to cuboidal peripheral cells with rare mitotic figures and central cells that may exhibit cystic degeneration, individual cell keratinization, or occasional calcification. Minor histological variations, including mucous metaplasia, sebaceous differentiation, or ghost cell–like areas, may also be observed. Importantly, SOT lacks key features typical of other odontogenic tumors, such as peripheral palisading, reverse nuclear polarity, stellate reticulum–like areas (WHO 2024). Cytological atypia, atypical mitoses, or cellular pleomorphism are also absent. Strict adherence to these diagnostic criteria represented a critical methodological aspect of the present review. Several previously reported cases were excluded because their histopathological descriptions were inconsistent with the current definition of SOT, particularly when features suggestive of other odontogenic tumors were reported. Because many historical reports predate the current WHO classification, all available histopathological microphotographs were independently reassessed by an experienced oral pathologist in order to minimize the risk of diagnostic misclassification.

Similarly, epithelial proliferations described as SOT‐like lesions arising within the walls of odontogenic cysts were excluded from the analysis. Although these proliferations may closely resemble SOT histologically, they are currently interpreted as reactive epithelial proliferations rather than true neoplastic lesions (WHO 2024). Failure to distinguish these entities may contribute to diagnostic ambiguity and potential overestimation of SOT frequency in earlier literature.

The differential diagnosis of SOT is particularly relevant in relation to acanthomatous ameloblastoma, which may exhibit prominent squamous differentiation (Pullon et al. 1975). In contrast to ameloblastoma, however, SOT lacks peripheral palisading of columnar cells, reverse nuclear polarity, and stellate reticulum‐like areas (Badni et al. 2012). Therefore, accurate diagnosis requires careful integration of histopathological findings with clinical and radiographic features.

Radiographically, the triangular radiolucent configuration has historically been considered a characteristic feature of SOT, particularly when located interproximally between adjacent tooth roots (Pullon et al. 1975). This appearance has been attributed to tumor growth along the periodontal ligament space, producing a wedge‐shaped radiolucency between adjacent roots. Although this configuration was frequently described in the included studies, substantial variability in the terminology used to characterize lesion morphology was observed (Doyle et al. 1977; Goldblatt et al. 1982; Cataldo et al. 1983; Yaacob 1990; Upadhyaya et al. 2020; Laungani et al. 2020). Because several reports referred to this pattern based on prior literature rather than explicit descriptions in the original case reports, radiographic morphology in the present review was classified exclusively according to the terminology reported by the authors. These observations suggest that the triangular radiographic pattern, although commonly described, should not be considered pathognomonic for intraosseous SOT.

Clinically, intraosseous SOT appears to exhibit greater heterogeneity than peripheral SOT. Multifocal presentation, recurrence, and occasional familial occurrence have been reported in the literature (Leider et al. 1989; Croonenborghs et al. 2020), whereas peripheral lesions consistently demonstrated a benign clinical course without recurrence in the available reports. Familial cases of multifocal SOT have been described in two studies (Leider et al. 1989; Croonenborghs et al. 2012). Leider et al. (1989) reported lesions affecting three siblings, suggesting possible familial aggregation, although one of these cases had previously been reported by McNeill et al. (1980). Later, Croonenborghs et al. (2020) described multifocal lesions in a mother and her son. Although these observations raise the possibility of hereditary predisposition, the available evidence remains limited and no germline mutation has yet been identified (WHO 2024).

Molecular findings further support the odontogenic epithelial origin of SOT. Siar et al. (2010) demonstrated immunoexpression of components of the NOTCH signaling pathway in the epithelial islands of SOT, suggesting a role in epithelial cytodifferentiation. In addition, Perdigão et al. (2004) reported strong expression of ameloblastin (AMBN), supporting the hypothesis that the tumor arises from odontogenic epithelial remnants such as dental lamina remnants or epithelial rests of Malassez. Nevertheless, further molecular studies are needed to better elucidate the biological mechanisms underlying this rare neoplasm.

Although SOT is generally considered a benign odontogenic tumor with limited growth potential, rare reports have described its association with SCC (Ide et al. 1999; Alotaibi et al. 2024). In both cases, the initial incisional biopsy demonstrated features of SOT, whereas examination of the complete surgical specimen revealed characteristic areas of SOT and SCC. These findings raise the possibility of malignant transformation. Therefore, careful histopathological evaluation and adequate sampling are essential when assessing lesions diagnosed as SOT.

These diagnostic considerations also have implications for treatment planning. In most reported cases, management consisted of conservative surgical approaches, such as local excision with or without curettage or extraction of the involved tooth (Reichart and Philipsen 1990; Badni et al. 2012). More extensive procedures, including bone resection, were performed less frequently and were generally reserved for larger or more aggressive lesions (Reichart and Philipsen 1990; Badni et al. 2012). Although recurrence appears to be uncommon, it has been reported in some cases, highlighting the importance of adequate surgical removal and appropriate clinical and radiographic follow‐up. In this context, careful histopathological evaluation and long‐term follow‐up remain advisable.

The present study has several methodological strengths. A comprehensive search strategy was applied across multiple databases and non‐indexed literature sources, and the risk of bias of the included studies was assessed using a validated tool. Furthermore, all available histopathological microphotographs were independently reassessed by an experienced oral pathologist to confirm the diagnostic features of SOT according to the WHO (2024) criteria. Nevertheless, some limitations must be acknowledged. The available evidence derives exclusively from case reports and small case series, reflecting the rarity of SOT and limiting the level of evidence that can be obtained. In addition, incomplete reporting of clinical, radiographic, and follow‐up information in several studies restricts the ability to draw more robust conclusions regarding the long‐term biological behavior of this tumor.

A previous review of SOT was conducted by Chrcanovic and Gomez (2018). However, that study included heterogeneous sources, such as narrative reviews, and did not follow PRISMA reporting guidelines. In contrast, the present review followed a registered protocol and applied stricter eligibility criteria based on the current WHO classification. The independent reassessment of case descriptions and histopathological microphotographs further contributed to improving diagnostic consistency across the included cases.

Taken together, the findings of this review reinforce the importance of strict diagnostic criteria and careful clinicopathological correlation for the accurate diagnosis of SOT. Increased awareness of its clinical, radiographic, and histopathological spectrum may improve differential diagnosis and help prevent misclassification with other odontogenic lesions. Given the rarity of SOT and the predominance of case‐based evidence, future multicenter studies and standardized case reporting are necessary to further clarify its biological behavior and optimal management.

## 5 Conclusion

SOT is a rare odontogenic neoplasm that may present diagnostic challenges due to its histopathological overlap with other odontogenic lesions, particularly acanthomatous ameloblastoma and SOT‐like epithelial proliferations associated with odontogenic cysts. Accurate diagnosis therefore requires strict adherence to contemporary WHO diagnostic criteria combined with careful clinicopathological correlation. Awareness of this entity among oral healthcare providers is essential to avoid potential misclassification and to guide appropriate management. Although SOT generally exhibits benign biological behavior, the possibility of recurrence and the rare reports of malignant transformation underscore the importance of appropriate surgical management and long‐term clinical follow‐up.

## Author Contributions

**Paula Sousa Lopes de Cascaes:** investigation, writing – original draft, methodology, writing – review and editing, data curation, formal analysis. **Licia Pacheco Teixeira:** investigation, methodology, validation. **Monikelly Carmo Narchini do Nascimento:** investigation, writing – review and editing, visualization, data curation. **Izabel Regina Fischer Rubira‐Bullen:** conceptualization, methodology, writing – review and editing, visualization. **José Luiz Cintra Junqueira:** conceptualization, methodology, writing – review and editing, visualization. **Mariana Quirino Silveira Soares:** conceptualization, supervision, visualization, formal analysis, validation, data curation. **Andresa Borges Soares:** conceptualization, methodology, writing – review and editing, validation, formal analysis, data curation.

## Funding

The authors have nothing to report.

## Ethics Statement

The authors have nothing to report.

## Consent

The authors have nothing to report.

## Conflicts of Interest

The authors declare no conflicts of interest.

## Supporting information


**Data S1:** Search strategies.
**Data S2:** PRISMA 2020 diagram.
**Data S3:** Individual analysis of the risk of bias of the included studies.
**Data S4:** Clinical, radiographic and histological characteristics of intraosseous SOT.
**Data S5:** Clinical, radiographic and histological characteristics of peripheral SOT.

## Data Availability

Synthesis of data collected from included studies is available in the Supporting Material.
