# Supplementary material for: Squamous Odontogenic Tumor: A Systematic Review
Source: Oral Dis. 2026 Apr 24;32(8):2206–19. doi: 10.1111/odi.70340 (PMC13693616; doi:10.1111/odi.70340)
Supplement: Supplementary file 1 [file ODI-32-2206-s001.docx]

| **Supplementary Material 1.** Search strategies. | | |
| --- | --- | --- |
| **Database or other source** | **Strategy** | **Records (April 2025)** |
| PubMed | “odontogenic tumor, squamous” OR "odontogenic tumors squamous"[All Fields] OR "squamous odontogenic tumors"[All Fields] OR "tumor squamous odontogenic"[All Fields] OR "tumors squamous odontogenic"[All Fields] OR "squamous odontogenic tumor"[All Fields] | 114 |
| Web of Science | “odontogenic tumor, squamous” OR “odontogenic tumors, squamous” OR “squamous odontogenic tumors” OR “tumor, squamous odontogenic” OR “tumors, squamous odontogenic” OR “squamous odontogenic tumor” (All Fields) | 98 |
| Cochrane | “odontogenic tumor, squamous” OR “odontogenic tumors, squamous” OR “squamous odontogenic tumors” OR “tumor, squamous odontogenic” OR “tumors, squamous odontogenic” OR “squamous odontogenic tumor” in All Text - (Word variations have been searched) | 0 |
| Scielo | “odontogenic tumors, squamous” OR “squamous odontogenic tumors” OR “tumor, squamous odontogenic” OR “tumors, squamous odontogenic” OR “squamous odontogenic tumor” | 2 |
| Embase | ‘odontogenic tumor, squamous’ OR ‘odontogenic tumors, squamous’ OR ‘squamous odontogenic tumors’ OR ‘tumor, squamous odontogenic’ OR ‘tumors, squamous odontogenic’ OR ‘squamous odontogenic tumor’ | 103 |
| Livivo | “odontogenic tumor, squamous” OR “odontogenic tumors, squamous” OR “squamous odontogenic tumors” OR “tumor, squamous odontogenic” OR “tumors, squamous odontogenic” OR “squamous odontogenic tumor” | 107 |
| Scopus | “odontogenic tumor, squamous” OR "odontogenic tumors, squamous" OR "squamous odontogenic tumors" OR "tumor, squamous odontogenic" OR "tumors, squamous odontogenic" OR "squamous odontogenic tumor" | 334 |
| BVS | “odontogenic tumor, squamous” OR "odontogenic tumors, squamous" OR "squamous odontogenic tumors" OR "tumor, squamous odontogenic" OR "tumors, squamous odontogenic" OR "squamous odontogenic tumor" AND instance:"lilacsplus" | 39 |
| IBICT/BDTD | '“odontogenic tumor, squamous” OR "odontogenic tumors, squamous" OR "squamous odontogenic tumors" OR "tumor, squamous odontogenic" OR "tumors, squamous odontogenic" OR "squamous odontogenic tumor"’ | 1 |
| Open Grey | “odontogenic tumor, squamous” OR “odontogenic tumors, squamous” OR “squamous odontogenic tumors” OR “tumor, squamous odontogenic” OR “tumors, squamous odontogenic” OR “squamous odontogenic tumor” | 110 |
| Pro Quest | “odontogenic tumor, squamous” OR “odontogenic tumors, squamous” OR “squamous odontogenic tumors” OR “tumor, squamous odontogenic” OR “tumors, squamous odontogenic” OR “squamous odontogenic tumor” | 224 |
| Google Scholar | “odontogenic tumor, squamous” OR “odontogenic tumors, squamous” OR “squamous odontogenic tumors” OR “tumor, squamous odontogenic” OR “tumors, squamous odontogenic” OR “squamous odontogenic tumor” | 100 |
| **Total** |  | **1,232** |

**Supplementary Material 2:** PRISMA 2020 diagram

**Supplementary material 3.** Individual analysis of the risk of bias of the included studies.

| Study | Q1 | Q2 | Q3 | Q4 | Q5 | Q6 | Q7 | Q8 | Risk of bias |
| --- | --- | --- | --- | --- | --- | --- | --- | --- | --- |
| Pullon et al., 1975 | ✓ | - | ✓ | ✓ | ✓ | ✓ | ✓ | ✓ | Low |
| Doyle et al., 1977 | ✓ | - | ✓ | ✓ | ✓ | - | - | ✓ | Moderate |
| McNeill et al., 1980 | ✓ | - | ✓ | ✓ | ✓ | ✓ | ✓ | ✓ | Low |
| Hopper et al., 1980 | ✓ | ✓ | ✓ | ✓ | ✓ | ✓ | ✓ | ✓ | Low |
| Carr et al., 1981 | ✓ | - | ✓ | ✓ | ✓ | - | - | ✓ | Moderate |
| Leventon et al., 1981 | - | ✓ | ✓ | - | ✓ | ✓ | ✓ | ✓ | Low |
| Goldblatt et al., 1982 | - | - | - | - | ✓ | - | - | ✓ | High |
| Cataldo et al., 1983 | ✓ | ✓ | ✓ | ✓ | ✓ | ✓ | ✓ | ✓ | Low |
| Norris et al., 1984 | - | ✓ | ✓ | ✓ | ✓ | ✓ | ✓ | ✓ | Low |
| Kristensen et al., 1985 | ✓ | ✓ | ✓ | ✓ | ✓ | ✓ | ✓ | ✓ | Low |
| Monteil & Terestri, 1985 | ✓ | ✓ | ✓ | ✓ | ✓ | ✓ | ✓ | ✓ | Low |
| Warnock et al., 1985 | ✓ | ✓ | ✓ | ✓ | ✓ | - | - | ✓ | Low |
| Hietanen et al., 1985 | ✓ | - | ✓ | - | ✓ | ✓ | ✓ | ✓ | Low |
| Tatemoto et al., 1989 | ✓ | - | ✓ | ✓ | ✓ | ✓ | ✓ | ✓ | Low |
| Leider et al., 1989 | ✓ | - | ✓ | ✓ | ✓ | - | - | ✓ | Moderate |
| Reichart & Philipsen, 1990 | - | ✓ | ✓ | ✓ | ✓ | ✓ | ✓ | ✓ | Low |
| Loyola et al., 1990 | ✓ | ✓ | ✓ | ✓ | ✓ | ✓ | ✓ | ✓ | Low |
| Yaacob, 1990 | ✓ | ✓ | ✓ | ✓ | ✓ | ✓ | ✓ | ✓ | Low |
| Baden et al., 1993 | - | ✓ | ✓ | ✓ | ✓ | ✓ | ✓ | ✓ | Low |
| Zak & Glickman, 1995 | ✓ | ✓ | ✓ | ✓ | ✓ | ✓ | ✓ | ✓ | Low |
| Favia et al., 1997 | - | ✓ | ✓ | ✓ | ✓ | ✓ | ✓ | ✓ | Low |
| Stoll et al., 1997 | ✓ | ✓ | ✓ | ✓ | ✓ | ✓ | ✓ | ✓ | Low |
| Kusama et al., 1998 | ✓ | ✓ | ✓ | ✓ | ✓ | ✓ | ✓ | ✓ | Low |
| Ide et al., 1999 | - | ✓ | ✓ | ✓ | ✓ | ✓ | ✓ | ✓ | Low |
| Ikai et al., 2001 | - | ✓ | ✓ | ✓ | ✓ | ✓ | ✓ | ✓ | Low |
| Gómez et al., 2001 | ✓ | ✓ | ✓ | ✓ | ✓ | ✓ | ✓ | ✓ | Low |
| Haghighat et al., 2002 | ✓ | ✓ | ✓ | ✓ | ✓ | ✓ | ✓ | ✓ | Low |
| Barrios et al., 2004 | ✓ | ✓ | ✓ | ✓ | ✓ | ✓ | ✓ | ✓ | Low |
| Cillo & Silverman, 2005 | ✓ | ✓ | ✓ | ✓ | ✓ | ✓ | ✓ | ✓ | Low |
| Iwaki et al., 2007 | - | ✓ | ✓ | ✓ | ✓ | ✓ | ✓ | ✓ | Low |
| Tamgadge et al., 2007 | ✓ | ✓ | ✓ | ✓ | ✓ | ✓ | ✓ | ✓ | Low |
| Siar et al., 2010 | ✓ | ✓ | ✓ | ✓ | ✓ | ✓ | ✓ | ✓ | Low |
| Jones et al., 2011 | - | ✓ | ✓ | ✓ | ✓ | ✓ | ✓ | ✓ | Low |
| Badni et al.,2012 | - | ✓ | ✓ | ✓ | ✓ | - | - | ✓ | Moderate |
| Malathi et al., 2012 | - | ✓ | ✓ | ✓ | ✓ | - | - | ✓ | Moderate |
| Bansal & Joshi, 2013 | - | ✓ | ✓ | ✓ | ✓ | ✓ | ✓ | ✓ | Low |
| Moussa et al., 2013 | - | ✓ | ✓ | ✓ | ✓ | - | - | ✓ | Moderate |
| Mohr, et al., 2015 | - | - | ✓ | ✓ | ✓ | ✓ | ✓ | ✓ | Low |
| Lucio et al., 2015 | ✓ | - | ✓ | ✓ | ✓ | ✓ | ✓ | ✓ | Low |
| Anjana et al., 2016 | - | ✓ | ✓ | ✓ | ✓ | ✓ | ✓ | ✓ | Low |
| Elmuradi et al., 2017 | ✓ | - | ✓ | ✓ | ✓ | - | - | ✓ | Moderate |
| Verhelst et al., 2018 | - | ✓ | ✓ | ✓ | ✓ | ✓ | ✓ | ✓ | Low |
| Mohanty et al., 2019 | - | ✓ | ✓ | ✓ | ✓ | - | - | ✓ | Moderate |
| Laungani et al., 2020 | - | ✓ | ✓ | ✓ | ✓ | ✓ | ✓ | ✓ | Low |
| Upadhyaya et al., 2020 | - | ✓ | ✓ | ✓ | ✓ | ✓ | ✓ | ✓ | Low |
| Croonenborghs et al., 2021 | ✓ | ✓ | ✓ | ✓ | ✓ | ✓ | ✓ | ✓ | Low |
| Cunha et al., 2023 | ✓ | ✓ | ✓ | ✓ | ✓ | ✓ | ✓ | ✓ | Low |
| Prasanth et al., 2023 | - | - | ✓ | ✓ | ✓ | ✓ | ✓ | ✓ | Low |
| Alotaibi et al., 2024 | - | ✓ | ✓ | ✓ | ✓ | - | - | ✓ | Moderate |
| Endo et al., 2024 | - | ✓ | ✓ | ✓ | ✓ | - | - | ✓ | Moderate |

**Supplementary Material 4.** Clinical, radiographic and histological characteristics of intraosseous SOT.

| **Author, country, year** | **Gender, age, ethnicity** | **Evolution time (months)** | **Clinical characteristics** | **Pain** | **Location** | **Size (mm)** | **Radiographic characteristics** | **Histopathological**  **characteristics** | **Treatment** | **Follow-up (months)** | **Recurrence** |
| --- | --- | --- | --- | --- | --- | --- | --- | --- | --- | --- | --- |
| Pullon et al., USA, 1975 | Female, 23 years, Afro-American | Not reported | Tooth Mobility | No | Multifocal  four quadrants | 40 | Shape Irregular  Borde smooth | Islands of squamous epithelium, fibrous connective stroma, cystic degeneration, areas of calcification, keratinized areas | Surgical excision | 5 | Yes |
| Pullon et al., USA, 1975 | Male, 11 years, Caucasian | 2 | Swelling | No | Anterior maxilla | 40 | Shape NR  impacted tooth | Islands of squamous epithelium, fibrous connective stroma, cystic degeneration | Surgical excision + currettage | 60 | No |
| Pullon et al., USA, 1975 | Male, 19 years, Caucasian | Incidental finding | Asymptomatic | No | Left  posterior mandible | 10 | Shape NR | Islands of squamous epithelium, fibrous tissue stroma, cystic degeneration | Surgical excision | 18 | No |
| Pullon et al., USA, 1975 | Female, 31 years, Caucasian | 4 | Tooth mobility | Not reported | Anterior and Posterior  Left  maxilla | Not reported | Shape NR  Divergence of roots | Islands of squamous epithelium, fibrous tissue stroma, areas of calcification | Surgical excision | 12 | No |
| Pullon et al., USA, 1975 | Female, 42 years, Caucasian | Not reported | Tooth mobility | Not reported | Anterior maxilla | Not reported | Shape NR | Islands of squamous epithelium, fibrous tissue stroma, cystic degeneration, calcified bodies | Surgical excision | 60 | No |
| Pullon et al., USA, 1975 | Male, 29 years, Caucasian | Not reported | Tooth mobility | No | Right posterior mandible | Not reported | Shape NR  impacted tooth | Islands of squamous epithelium, fibrous tissue stroma, cystic degeneration | Surgical excision | 216 | No |
| Doyle et al., USA, 1977 | Male, 26 years, Afro-American | Not reported | Tooth mobility | Yes | Anterior and  Posterior  Right  maxilla | Not reported | Shape Irregular | Islands of squamous epithelium, fibrous tissue stroma, cystic degeneration, calcifications | Surgical excision + tooth extraction | Not reported | Not reported |
| Doyle et al., USA, 1977 | Male, 65 years, not reported | Not reported | Swelling | No | Right posterior maxilla | Not reported | Shape NR  “Soap bubble”  multilocular | Islands of squamous epithelium, fibrous tissue stroma, area of calcification | Bone resection + tooth extraction | 7 | No |
| McNeil et al., USA, 1980 | Female, 26 years, Afro-American | Not reported | Pain | Yes | Multifocal  four quadrants | Not reported | severe bone loss | Islands of squamous epithelium, fibrous tissue stroma, cystic degeneration, microcalcifications, rare keratinizations | Surgical excision+ tooth extraction | 12 | Yes |
| Hopper et al., USA, 1980 | Female, 22 years, Caucasian | 12 | Tooth mobility | No | Multifocal  left  posterior | 70 | Shape NR  Irregular | Islands of squamous epithelium, fibrous tissue stroma, cystic degeneration, calcified areas, rare keratinization | Bone  Resection+ tooth extraction (maxilla)  Surgical excision (mandibule) | 3 | No |
| Carr et al., USA, 1981 | Female, 66 years, Caucasian | 3 | Swelling + tooth mobility | No | Left  posterior maxilla | Not reported | Shape Triangular  root resorption  root divergence | Islands of squamous epithelium, fibrous tissue stroma, cystic degeneration, keratinization, no calcification | Surgical excision+ tooth extraction | Not reported | Not reported |
| Leventon et al., USA, 1981 | Male, 59 years, Caucasian | Incidental finding | Asymptomatic | No | Left  posterior mandible | 15 | Shape NR | Islands of squamous epithelium, fibrous tissue stroma, cystic degenerations | Surgical excision | 7 | No |
| Goldblatt et al., USA, 1982 | Female, 60 years, not reported | Not reported | Pain, drainage, tender to  percussion | Yes | Right  Posterior Maxilla | 2 | Shape ovoid | Islands of squamous epithelium, fibrous tissue stroma, cystic degenerations | Tooth extraction +  Curettage | Not reported | Not reported |
| Goldblatt et al., USA, 1982 | Male, 29 years, not reported | Not reported | Discomfort | Not reported | Right  Posterior Maxilla | Not reported | Shape hemispherical | Islands of squamous epithelium, fibrous tissue stroma, microcystic degenerations, rare keratinizations | Surgical excision | Not reported | Not reported |
| Goldblatt et al., USA, 1982 | Female, 30 years, Caucasian | Not reported | Tooth mobility | Not reported | Left  Posterior  mandible | 3 | Shape NR | Islands of squamous epithelium, fibrous tissue stroma, microcystic degenerations, | Tooth extraction +  Curettage | Not reported | Not reported |
| Goldblatt et al., USA, 1982 | Female, 26 years, Caucasian | Not reported | Not reported | Not reported | Posterior mandible  Side not reported | 2 | Shape triangular | Islands of squamous epithelium, fibrous tissue stroma, microcystic degenerations, | Curettage | Not reported | Yes |
| Goldblatt et al., USA, 1982 | Female, 67 years, Caucasian | Not reported | Not reported | Not reported | Posterior and anterior  Not reported | Not reported | Not reported | Islands of squamous epithelium, fibrous tissue stroma, microcystic degenerations, | Surgical excision | Not reported | Not reported |
| Cataldo et al., USA, 1983 | Female, 24 years, Caucasian | Not reported | Thermal sensitivity | No | Left  posterior  maxilla | 45 | Shape Pear-shaped / scalloped  root divergence | Islands of squamous epithelium, fibrous tissue stroma, microcystic degenerations, calcifications, keratinization | Surgical excision | 8 | No |
| Norris et al., USA, 1985 | Male, 26 years, Afro-American | 2 | Swelling | Yes | Bilateral Maxilla  Posterior | Not reported | Shape NR  impacted tooth, | Islands of squamous epithelium, fibrous tissue stroma, areas keratinization, | Surgical excision | No | Died |
| Kristensen et al., Denmark, 1985 | Male, 61 years, not reported | 3 | Swelling | Yes | Anterior maxilla | Not reported | Shape NR | Islands of squamous epithelium, fibrous tissue stroma, keratinizations, microcysts | Bone resection | 84 | No |
| Monteil & Terestri, France, 1985 | Female, 51 years, Caucasian | Not reported | Swelling+ paresthesia | No | Posterior  Left  mandible | 35 | Shape oval  unilocular  impacted tooth | Islands of squamous epithelium, fibrous tissue stroma, microcystic degenerations, calcifications | Surgical excision Tooth extraction + | 9 | No |
| Warnock et al., USA, 1985 | Male, 19 years, Afro-American | Incidental finding | Asymptomatic | No | Posterior  Right  mandible | 10 | Shape Triangular | Islands of squamous epithelium,  stroma of fibrous tissue, microcystic degenerations | Surgical excision + curettage | Not reported | Not reported |
| Tatemoto et al., Japan, 1989 | Male, 41 years, not rerported | No | Swelling | Not reported | Anterior mandible | 5 | Shape NR | Islands of squamous epithelium, fibrous tissue stroma, keratinization cellular | Surgical excision + Tooth extraction | Not reported | Not reported |
| Tatemoto et al., Japan, 1989 | Male, 56 years, not rerported | No | Swelling | Not reported | Anterior maxilla | Not reported | Shape Irregular | Islands of squamous epithelium, fibrous tissue stroma, keratinization | Bone Resection + Tooth extraction | Not reported | Not reported |
| Leider et al., USA, 1989 | Male, 29 years, Afro-American | Incidental finding | Asyntomatic | No | Multifocal maxilla mandible | Not reported | Shape NR  Multilocular | Islands of squamous epithelium, fibrous tissue stroma, microcystic degenerations, calcifications | Tooth extraction + Curettage | Yes | Not reported |
| Leider et al., USA, 1989 | Male, 25 years, Afro-American | Not reported | Swelling | No | Multifocal maxilla mandible | 20 | Multiple radiolucent | Islands of squamous epithelium, fibrous tissue stroma | Tooth extraction + curettage | Yes | No |
| Reichart & Philipsen,Germany, 1990 | Female, 56 years, Turkish | 3 | Tooth Mobility + Swelling | Yes | Right  posterior  mandible | Not reported | Shape NR  Multilocular  divergence of roots | Islands of squamous epithelium, fibrous tissue stroma, microcystic degenerations, | Tooth extraction + Bone resection | 12 | No |
| Loyola et al., Brazil, 1990 | Female, 37 years, not reported | Incidental finding | Swelling | No | Right  posterior  maxilla | Not reported | Shape NR  impacted tooth | Islands of squamous epithelium, fibrous tissue stroma, microcystic degenerations, | Surgical excision + Tooth extraction | 7 | No |
| Yaacob, Malaysia, 1990 | Male, 39 years, Asian | Not reported | Swelling | No | Right  posterior  maxilla | 30 | Shape hemispherical | Islands of squamous epithelium, fibrous tissue stroma, microcystic degenerations, keratinization, calficication | Surgical excision | 48 | No |
| Baden et al., France, 1993 | Male, 46 years,not reported | Not reported | Swelling + ulceration | No | Right  posterior  maxilla | Not reported | Shape NR | Islands of squamous epithelium, fibrous tissue stroma, microcystic degenerations, keratinization | Surgical excision | 84 | No |
| Baden et al., France, 1993 | Male, 39 years, not reported | Not reported | Swelling | No | Left  posterior  mandible | 15 | Shape NR  Multilocular | Islands of squamous epithelium, fibrous tissue stroma, microcystic degenerations, keratinization | Bone resection | 12 | No |
| Zak & Glickman, USA, 1995 | Female, 28 years, Caucasian | Incidental finding | Tooth Mobility | No | Anterior maxilla | Not reported | Shape NR | Islands of squamous epithelium, fibrous tissue stroma | Bone resection + Tooth extraction | 12 | No |
| Favia et al., Italy, 1997 | Not reported, 56 years, not reported | 3 | Swelling | Yes | Right  posterior maxilla | 30 | Shape NR  Unilocular | Islands of squamous epithelium, fibrous tissue stroma, microcystic degenerations and keratin pearls | Surgical excision | 60 | No |
| Favia et al., Italy, 1997 | Female, 25 years, not reported | Not reported | Gingival recession | No | Rigth  posterior mandible | Not reported | Shape NR | Islands of squamous epithelium, fibrous tissue stroma, microcystic degenerations, Keratinisation | Surgical excision | 72 | No |
| Stoll et al., Germany, 1997 | Male, 53a, Caucasian | 24 | Swelling + Tooth Mobility | No | Left  posterior mandible | 55 | Shape NR  Unilocular  root resorption displacement  mandibular canal | Islands of squamous epithelium, fibrous tissue stroma, microcystic degenerations, | Surgical excision+ Tooth extraction | 30 | No |
| Kusama et al., Japan, 1998 | Female, 42a, Asian | 2 | Swelling | Yes | Left  posterior maxilla | Not reported | Shape NR  unilocular  impacted tooth | Islands of squamous epithelium, fibrous tissue stroma, microcystic degenerations, | Surgical excision+ Tooth extraction | 20 | No |
| Ide et al., Japan, 1999 | Male, 53 years, not reported | 60 | Swelling | No | Left  posterior mandible | 15 | Shape NR  Unilocular  impacted tooth | Islands of squamous epithelium, fibrous tissue stroma, microcystic degenerations | Surgical excision+ Tooth extraction | 2 | Yes |
| Gomez et al., USA, 2001 | Female, 30 years, Afro-American | Incidental finding | Asymptomatic | No | Anterior maxilla | 20 | Shape Triangular  unilocular  divergent roots | Islands of squamous epithelium, fibrous tissue stroma, microcystic degenerations, | Surgical excision + curettage | Not reported | Not reported |
| Haghighat et al., USA, 2002 | Male, 43 years, Afro-American | Not reported | Gingival recession | No | Anterior maxilla | 18 | Shape NR  divergent roots | Islands of squamous epithelium, fibrous tissue stroma, microcystic degenerations, keratinization | Surgical excision + curettage | 18 | No |
| Barrios et al., USA, 2004 | Male, 11 years, Caucasian | 4 | Swelling | Yes | Anterior maxilla | Not reported | No radiographic alteration | Islands of squamous epithelium, fibrous tissue stroma, keratinization | Surgical excision + curettage | 28 | No |
| Cillo & Silverman, USA, 2005 | Female, 45 years, Afro-American | Incidental finding | Asymptomatic | No | Left  posterior  mandible | Not reported | Shape NR  impacted tooth | Islands of squamous epithelium, fibrous tissue stroma | Surgical excision+ Tooth extraction+ curettage | 4 | No |
| Iwaki et al., Japan, 2007 | Male, 50 years, not reported | Not reported | Swelling + ulceration | No | Right  posterior  maxilla | Not reported | Shape NR | Islands of squamous epithelium, fibrous tissue stroma, microcystic degenerations, keratinization, calcifications | Bone resection+ Tooth extraction | Yes | Not reported |
| Tamgadge et al., India, 2007 | Male, 37 years, not reported | 6 | Swelling | No | Anterior maxilla | 25 | Shape triangular  unilocular | Islands of squamous epithelium, fibrous tissue stroma | Surgical excision | Not reported | Not reported |
| Siar et al., Japan, 2010 | Female, 10 years, Asian | Not reported | Swelling | No | Anterior mandible | Not reported | Not reported | Islands of squamous epithelium, fibrous stroma, microcystic degenerations, keratinization, calcifications, | Surgical excision | Not reported | No |
| Jones et al., USA, 2011 | Male, 24 years, not reported | Incidental finding | Swelling | No | Anterior maxilla | Not reported | Shape triangular  unilocular  divergent roots | Islands of squamous epithelium, fibrous tissue stroma, microcystic degenerations, calcification | Bone resection+ Tooth extraction | Not reported | Not reported |
| Badni et al., India, 2012 | Male, 58 years, not reported | 3 | Swelling + Tooth mobility | No | Anterior mandible | Not reported | Shape Triangular  divergent roots | Islands of squamous epithelium, fibrous tissue stroma, | Surgical excision | Not reported | Not reported |
| Bansal & Joshi, India, 2013 | Female, 26 years, not reported | 11 | Swelling + Tooth mobility | No | Anterior mandible | Not reported | Shape Triangular  divergent roots | Islands of squamous epithelium, fibrous tissue stroma, microcystic degenerations | Surgical excision+ curettage | 18 | No |
| Moussa et al., Egypt, 2013 | Female, 15 years, not reported | 3 | Swelling + Tooth mobility | No | Anterior maxilla | Not reported | Shape NR | Islands of squamous epithelium, fibrous tissue stroma, microcystic degenerations, | Surgical excision | Not reported | Not reported |
| Mohr et al., Germany, 2015 | Female, 41 years, not reported | 11 | Sensitivity+ Swelling | No | Anterior maxilla | Not reported | Shape Triangular  divergent roots | Islands of squamous epithelium, fibrous tissue stroma, microcystic degenerations | Surgical excision | 72 | Yes  2x |
| Lúcio et al., Brazil, 2015 | Female, 56 years, Afro-American | Not reported | Swelling | Not reported | Anterior mandible | 37.25 | Shape NR  Multilocular | Islands of squamous epithelium, fibrous tissue stroma, microcystic degenerations, | Surgical excision+ curettage | 50 | No |
| Elmuradi et al., USA, 2017 | Male, 43 years, Afro-American | Not reported | Swelling | Not reported | Multifocal  Maxila  mandible | Not reported | Multiple radiolucent areas | Squamous epithelium islands,  fibrous tissue stroma, microcystic degenerations, calcification | Bone resection + Tooth extraction + curettage | No | No |
| Verhelst et al., Belgium, 2018 | Female, 29 years, not reported | 3 | Swelling + Tooth mobility | No | Multifocal  Maxila  mandible | Not reported | Triangular radiolucency displacement roots | Squamous epithelium islands,  fibrous tissue stroma, | Surgical excision+ curettage | Yes | Yes |
| Mohanty et al., India, 2019 | Male, 38 years, not reported | 60 | Swelling | No | Anterior maxilla | Not reported | Shape NR  displacement root  root resorption | Squamous epithelium islands,  fibrous tissue stroma, cystic degeneration, | Surgical excision | 12 | No |
| Laugani et al., USA, 2020 | Female, 19 years, not reported | 4 and ½  (4.5) | Sensitivity+ Swelling | No | Right  posterior mandible | 10 | Teardrop-shaped  impacted tooth | Squamous epithelium islands,  fibrous tissue stroma, cystic degeneration, | Surgical excision+ Tooth extraction | Yes | No |
| Upadhyaya et al., USA, 2020 | Female, 44 years, not reported | ¾  (0.75) | Pain | Yes | Right  posterior mandible | 18 | Shape band-like  divergence of roots | Squamous epithelium islands,  fibrous tissue stroma, cystic degeneration, keratinization | Tooth extraction + curettage | 1 | No |
| Croonenborghs et al., Belgium, 2021 | Male, 14 years, Caucasian | 4.5 | Swelling | No | Multifocal  Anterior  posterior maxilla | Not reported | Shape Triangular root divergence | Squamous epithelium islands,  fibrous tissue stroma, cystic degeneration | Bone resection + Surgical excision | 24 | No |
| Cunha et al., Brazil, 2023 | Male, 25 years, Afro-American | 12 | Swelling +  tooth mobility | No | Left  posterior  maxilla | 45 | Shape Triangular  Unilocular  impacted tooth divergente roots | Squamous epithelium islands,  fibrous tissue stroma, cystic degeneration, keratinization, | Surgical excision | 12 | No |
| Alotaibi et al., USA, 2024 | Male, 64 years, not reported | 24 | Fistula+ trismus | Not reported | Right  Posterior  maxilla | Not repórter | Shape NR | Squamous epithelium islands,  fibrous tissue stroma | Bone resection + Surgical excision | Not reported | Not reported |
| Endo et al., USA,  2024 | Female, 25 years, not reported | Not reported | Swelling | No | Right  posterior  maxilla | Not reported | Shape NR  unilocular | Squamous epithelium islands,  fibrous tissue stroma, cystic degeneration, calcifications | Surgical excision | Not reported | Not reported |

**Supplementary Material 5.** Clinical, radiographic and histological characteristics of peripheral SOT

| **Author, country, year** | **Gender, age, ethnicity** | **Evolution time** | **Clinical characteristics** | **Pain** | **Location** | **Size (mm)** | **Radiographic characteristics** | **Histopathological characteristics** | **Treatment** | **Follow-up (months)** | **Recurrence** |
| --- | --- | --- | --- | --- | --- | --- | --- | --- | --- | --- | --- |
| Hietanen et al.,Finland, 1985 | Female, 11 years, not reported | Not reported | Swelling | Not reported | Anterior maxilla | 5 | No bone destruction (extra-osseous lesion) | Squamous epithelium islands,  fibrous tissue stroma, cystic degeneration, calcification, keratinization | Surgical excision | 6 | No |
| Baden et al., France, 1993 | Male, 74 years, not reported | Not reported | Swelling | Not reported | Maxilla  Region not reorted | Not reported | Normal | Squamous epithelium islands,  fibrous tissue stroma, cystic degeneration | Surgical excision | 15 | No |
| Ikai et al., Japan, 2001 | Male, 30 years, not reported | 12 | Swelling | No | Anterior mandible | 10 | No bone resorption | Squamous epithelium islands,  fibrous tissue stroma, cystic degeneration, calcification | Tooth extraction+ Surgical excision | 12 | No |
| Malathi et al., India,  2012 | Female, 60 years, not reported | 24 | Swelling | Not reported | Right posterior  mandible | 20 | Loss of interdental bone at the site of the lesion | Squamous epithelium islands,  fibrous tissue stroma, cystic degeneration, keratinization | Surgical excision | Not reported | Not reported |
| Anjana et al, India, 2016 | Male, 59 years, not reported | 24 | Swelling | No | Anterior maxilla | 25 | No change | Squamous epithelium islands,  fibrous tissue stroma, cystic degeneration | Surgical excision | 5 | No |
| Prasanth et al., India, 2023 | Male, 29 years, not reported | Not reported | Pain | Yes | Left posterior mandible | 15 | Impacted tooth | Squamous epithelium islands,  fibrous tissue stroma | Surgical excision | 12 | No |
